# Alzheimer's Disease Detection in Brain Magnetic Resonance Images Using Multiscale Fractal Analysis

**DOI:** 10.5402/2013/627303

**Published:** 2013-10-29

**Authors:** Salim Lahmiri, Mounir Boukadoum

**Affiliations:** Department of Computer Science, University of Quebec at Montreal, 201 President-Kennedy, Local PK-4150, Montreal, QC, Canada H2X 3Y7

## Abstract

We present a new automated system for the detection of brain magnetic resonance images (MRI) affected by Alzheimer's disease (AD). The MRI is analyzed by means of multiscale analysis (MSA) to obtain its fractals at six different scales. The extracted fractals are used as features to differentiate healthy brain MRI from those of AD by a support vector machine (SVM) classifier. The result of classifying 93 brain MRIs consisting of 51 images of healthy brains and 42 of brains affected by AD, using leave-one-out cross-validation method, yielded 99.18% ± 0.01 classification accuracy, 100% sensitivity, and 98.20% ± 0.02 specificity. These results and a processing time of 5.64 seconds indicate that the proposed approach may be an efficient diagnostic aid for radiologists in the screening for AD.

## 1. Introduction

Alzheimer's disease (AD) is a progressive and degenerative disease that affects brain cells, and its early diagnosis has been essential for appropriate intervention by health professionals. Noninvasive *in vivo* neuroimaging techniques such as magnetic resonance imaging (MRI) and positron emission tomography (PET) are commonly used to diagnose and monitor the progression of the disease and the effect of treatment. In this regard, the problem of developing computer aided diagnosis (CAD) tools to distinguish images with AD from those of normal brains has been extensively addressed in the past years [1–12]. A review of a recent work follows.

Magnin et al. [1] used the relative weight of gray matter versus white matter and cerebrospinal fluid in 90 regions of interests (ROI) as features classified with SVM. Based on the bootstrap method, the SVM obtained 94.5% average classification accuracy in the classification of 16 AD and 22 control (healthy) subjects, with a mean specificity of 96.6% and a mean sensitivity of 91.5%. Ramírez et al. [2] proposed a classification system for AD based on the partial least square (PLS) regression model for feature extraction (identification of discriminative voxels) and the random forest (RF) classifier. The PLS-RF system yielded accuracy, sensitivity, and specificity values of 96.9%, 100%, and 92.7%, respectively, after classifying 41 normal and 56 AD images using the leave-one-out cross-validation method. Salas-Gonzalez et al. [3] used Welch's *t*-test to identify voxels that provide higher difference between normal and AD images. The identified voxels formed the main features to classify, and a SVM with linear kernel reached 96.2% accuracy in distinguishing 41 normal and 38 AD images using leave-one-out cross-validation method; the sensitivity and the specificity were about 96%. Chincarini et al. [4] sampled the brain with seven relatively small volumes that were filtered to give intensity and textural MRI-based features. Each filtered region was analyzed with a random forest classifier to extract relevant features, which were subsequently processed with a support vector machine. The system performance was evaluated on the classification of 144 AD patients and 189 controls. Using receiver operating curve (ROC) analysis for 144 AD patients and 189 controls, and 20-fold cross-validation, the result was 0.97 area under curve (AUC) for discriminating the AD images from the normal ones, with 89% sensitivity and 94% specificity. Chen et al. [5] used large-scale network (LSN) analysis to classify subjects with Alzheimer's disease and normal subjects. The Pearson product moment correlation coefficients were used to assess the connectivity between ROI in the brain. Classification of the AD group (20 subjects) and the non-AD group (20 subjects) using leave-one-out cross-validation led to 87% AUC, 85% sensitivity, and 80% specificity. Graña et al. [6] used fractional anisotropy of diffusion tensor imaging (DTI) data to obtain information about the magnitude of the water diffusion process at each voxel. Based on leave-one-out cross-validation method, the linear kernel SVM achieved overall accuracy of 100% for the classification of 25 healthy controls and 20 AD images. Wolz et al. [7] used the hippocampal volume (HV), cortical thickness (CTH), tensor-based morphometry (TBM), and features extracted from manifold-based learning (MBL) framework to discriminate healthy controls (231 subjects) from subjects with AD (198 subjects). Five percent of the evaluation subjects were kept for testing and the remaining 95% were used for training. The SVM achieved 86% correct classification rate, 94% sensitivity, and 78% specificity. Zhang et al. [8] proposed a multimodal data fusion and classification method based on features extracted from structural MRI, functional imaging (FDG-PET), and cerebrospinal fluid (CSF). The three modalities were, respectively, used to measure brain atrophy and to quantify hypometabolism and specific proteins linked to AD. Consequently, 93 volumetric features were extracted from 93 ROI automatically labeled by an atlas warping algorithm. The linear SVM was used to evaluate the classification accuracy using 10-fold cross-validation. The result of classifying 51 AD and 52 normal controls yielded classification accuracy of 93.2%, with a sensitivity of 93% and a specificity of 93.3%. Daliri [9] used the scale-invariant feature transforms (SIFT) to extract features from MR images that were clustered using the *K*-means algorithm. Fisher's discriminant ratio was used for ranking clustered features, and genetic algorithms performed feature subset selection. The validation data consisted of the MRIs from 98 normal subjects and from 100 subjects with AD. The SVM achieved 86% correct classification rate using leave-one-out cross-validation method. Gray et al. [10] combined cross-sectional and longitudinal FDG-PET information for classification. Particularly, the whole brain was segmented into 83 anatomically defined regions from which intensities and changes in signal intensity over the follow-up period were extracted. The SVM achieved a classification accuracy of 88% for 200 patients with Alzheimer's disease and 200 healthy controls using fivefold cross-validation protocol. Li et al. [11] used longitudinal changes of cortical thickness to characterize AD pathology. In particular, three categories of features were extracted from each subject, including static cortical thickness, cortex thinning dynamics, and the correlation between the longitudinal thickness changes of different regions of interest (ROI) in brain image. Based on leave-one-out cross-validation method, the SVM distinguished 37 AD patients from 40 NC with an accuracy of 96.1%.

In a recent work, we proposed a fractal-based processing methodology to detect AD in brain MRI [12]. The system does not require image reduction or segmentation, and it relies on a simple three-step algorithm. First, the brain MRI is transformed into a one-dimensional (1D) signal by row concatenation. Then, a three-component feature vector is extracted from the 1D signal to characterize its local and global fractal features as expressed by Hurst's exponent and the two results from the detrended fluctuation analysis (DFA) [13] of the cumulated 1D signal: the scaling exponent and the total detrended fluctuation energy (Hurst's exponent allows the evaluation of how a signal is self-affine, i.e., can be made self-similar by an affine transformation for a given level of detail (a self-similar image is one whose whole is similar to its parts); DFA is a generalization that can also detect long-range power-law correlations in seemingly nonstationary signals [13]. In particular, it can determine local trends in the signal and measure its level of persistence). In the last step, the obtained feature vector is classified by a SVM with polynomial kernel. The validation with 10 normal brain MRI and 13 corresponding to AD led to 100% classification accuracy by a SVM with quadratic kernel. The obtained results suggested that the use of fractals to characterize the MRI of normal patients and ones with AD held the promise of equal or better classification accuracy than the best alternative approach while being simpler to implement.

The perfect classification accuracy reported in [12] was encouraging. However, it was achieved with a small set of MRI for validation. In addition, the average processing time to extract the fractal features took over 400 seconds. This paper follows up the work in [12] with a faster algorithm and a bigger database for validation. First, the features extraction processing time is reduced considerably by using multiscale analysis (MSA) [14] as alternative for Hurst's and DFA exponent estimation. Indeed, the approach reported by Lahmiri and Boukadoum in [12] determines the sought exponents by running polynomial regressions on multiple boxes or intervals of the 1D signal; on the other hand, MSA uses the generalized Hurst's exponent method [14], which determines the scaling properties of the signal by computing the *q*th-order moments of the distribution of the signal's increments [14]. This method combines the sensitivity to any type of signal dependence with a high computational efficiency due to its simple algorithm [14]. As each scale *q* allows the estimation of a Hurst's exponent, varying this value allows obtaining the multifractals of the signal in a straightforward way. 

An additional contribution of this work is the use of a relatively larger database for validation in comparison to [12]. Finally, our approach does not require a predetermined region of interest as done in [1, 5, 8, 10] and avoids extracting a large number of features to characterize the image in different modalities as done in [3, 4, 6–9, 11]. Instead, the multifractal analysis is performed on the whole brain MR image, and the extracted fractals are used to distinguish AD images from normal ones by the support vector machine. The SVM is a pattern recognition technique based on the statistical learning theory that finds the optimal nonlinear hyperplane that minimizes the expected classification error [15]. It was successfully applied in previous works [1, 3, 6, 8–12]. 

The balance of this paper is as follows.  Section 2 presents the multiscale analysis used to obtain the generalized Hurst's exponents used to characterize brain MR images and the SVM used for classification. In Section 3, we describe the obtained classification performance in terms of the accuracy rate, sensibility, and sensitivity. Finally, our conclusions are drawn in Section 4.

## 2. Material and Methods 

### 2.1. Image Database

**Figure 1:**
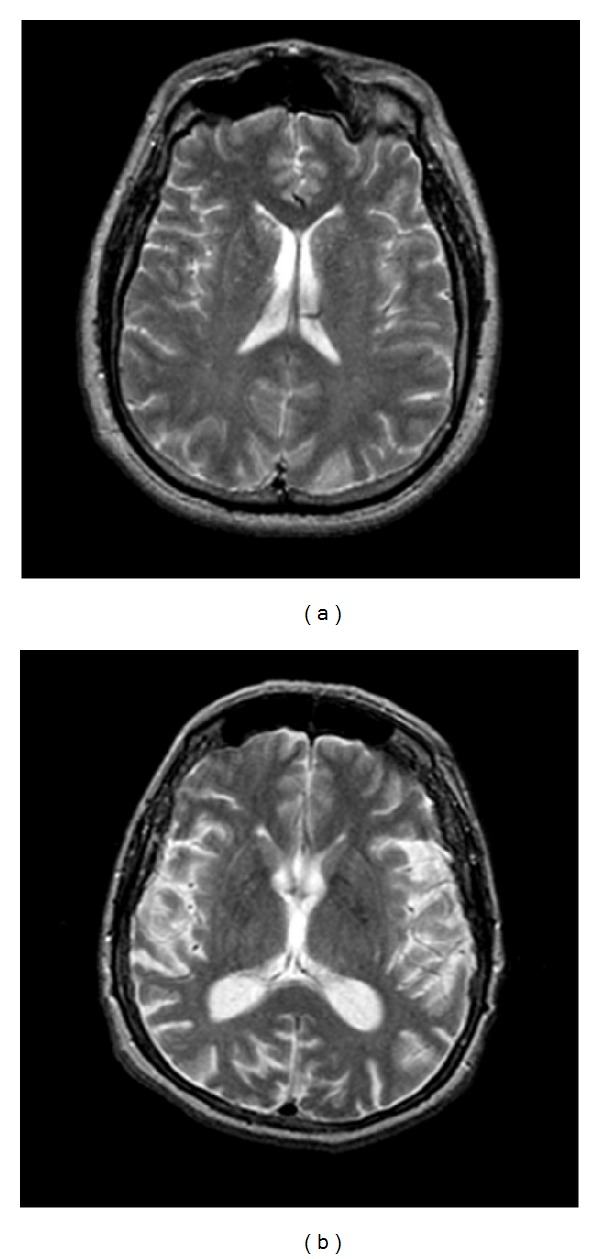
Healthy image (a) and AD image (b) in grayscale.

A collection of 93 axial, T2-weighted MR brain images of 256 × 256 size were downloaded from the Harvard Medical School webpage [16]. The set included 51 images of normal (healthy) brains and 42 of abnormal (unhealthy) brains affected by AD. We notice that there is no indication in the database regarding the AD stage.  Figure 1 shows examples of healthy and AD images used in the experiments. 

### 2.2. Multiscaling Analysis

Consider a signal *S*(*t*) defined at discrete time intervals *t* = *v*, 2*v*,…, *T* over a period *T* that is an integer multiple of *v*. The *q*th-order moments of the distribution that characterize the statistical evolution of *S*(*t*) are defined as follows [17]:

(1) $$\begin{array}{c} K_{q}\left(d\right)=\frac{\langle {||S\left(t+d\right)-S\left(t\right)||}^{q}\rangle }{\langle {\left|S\left(t\right)\right|}^{q}\rangle }, \end{array}$$

where *d* ∈ [*v*,  *d*_max⁡_] is a time interval and *d*_max⁡_ is its predetermined upper limit. The generalized Hurst's exponent *H*(*q*) is defined from the scaling behavior of *K*_*q*_(*d*) according to the following empirical relation [17]:

(2) $$\begin{array}{c} K_{q}\left(d\right)\propto {\left(\frac{d}{v}\right)}^{qH(q)}. \end{array}$$

If *K*_*q*_(*d*) and *d* satisfy a linear relationship for a given order *q* in log-log scale, Hurst's exponent *H*(*q*) can be estimated by running a linear regression of log⁡(*K*_*q*_(*d*)) versus log⁡(*d*). The generalized Hurst's exponent *H*(*q*) describes the long-memory dependence or persistence in the signal *S*(*t*). The multiscaling structure of signal *S*(*t*) is related to different orders *q* of the exponent *H*(*q*). In general, when *H*(*q*) > 0.5, the signal fluctuations related to the order *q* are persistent. When *H*(*q*) < 0.5, the signal fluctuations related to order *q* are antipersistent. Finally, the signal fluctuations are those of a random walk if *H*(*q*) = 0.5 [17]. Notice that *H*(*q* = 2) corresponds to the classic Hurst's exponent [14]. 

In this paper, the range of *q* is arbitrarily fixed to the interval from 1 to 6. Higher moments could also have been considered as will be discussed in Section 4. 

The original MRI is transformed into a one-dimensional (1D) signal by row concatenation. Then, Hurst's exponents *H*(*q*) for *q* = 1,…, 6 are estimated by applying the MSA algorithm. The resulting six-component feature vector forms the input of the SVM classifier to perform the identification of AD images. 

### 2.3. The Support Vector Machine Classifier

Introduced by Vapnik [15], the support vector machine (SVM) classifier is based on statistical learning theory. It implements the principle of structural risk minimization and has excellent generalization ability as a result, even when the data sample is small. The SVM performs a classification tasks by constructing an optimal separating hyperplane that maximizes the margin between the two nearest data points belonging to two separate classes. Given a training set {(*x*_*i*_, *y*_*i*_), *i* = 1,2,…, *m*}, where the input *x*_*i*_ ∈ *R* ^*d*^ and class labels *y*_*i*_ ∈ {+1, −1}, the separation hyperplane for a linearly separable binary classification problem is given by

(3) $$\begin{array}{c} f\left(x\right)=\langle w\cdot x\rangle +b, \end{array}$$

where *w* is a weight vector and *b* is a bias. The optimal separation hyperplane is found by solving the following optimization problem:

(4) $$\begin{array}{c} \underset{w,b,\xi }{\text{Minimize}}\;\frac{1}{2}\langle w\cdot w\rangle +C\overset{m}{\underset{i=1}{\sum }}\xi_{i} \end{array}$$

subject to

(5) $$\begin{array}{c} y_{i}\left(\langle w\cdot x_{i}\rangle +b\right)+\xi_{i}-1\geq 0,\;\xi_{i}\geq 0, \end{array}$$

where *C* is a penalty parameter that controls the tradeoff between the complexity of the decision function and the number of misclassified training examples and *ξ* is a positive slack variable. The previous optimization model can be solved by introducing Lagrange multipliers and using the Karush-Kuhn-Tucker theorem of optimization to obtain the solution as

(6) $$\begin{array}{c} w=\overset{m}{\underset{i=1}{\sum }}\alpha_{i}y_{i}x_{i}. \end{array}$$

The *x*_*i*_ values corresponding to positive Lagrange multipliers *α*_*i*_ are called support vectors, and they define the decision boundary. The *x*_*i*_ values corresponding to zero *α*_*i*_ are irrelevant. Once the optimal values of *α*, α* are found, the optimal hyperplane parameters w* and b* are determined. Then, the discriminant function of the SVM for a linearly separable binary classification problem is given by

(7) $$\begin{array}{c} g\left(x\right)=\mathrm{sign}\left(\overset{m}{\underset{i=1}{\sum }}y_{i}\alpha_{i}^{*}\langle x_{i}\cdot x\rangle +b^{*}\right). \end{array}$$

In the nonlinearly separable case, the SVM classifier nonlinearly maps the training points to a high dimensional feature space using a kernel function Φ, where linear separation can be possible. The scalar product 〈Φ(*x*_*i*_) · Φ(*x*_*j*_)〉 is computed by Mercer kernel function *K* as *K*(*x*_*i*_, *x*_*j*_) = 〈Φ(*x*_*i*_) · Φ(*x*_*j*_)〉. Then, the nonlinear SVM classifier has the following form:

(8) $$\begin{array}{c} g\left(x\right)=\mathrm{sign}\left(\overset{m}{\underset{i=1}{\sum }}y_{i}\alpha_{i}^{*}K\langle x,x_{i}\rangle +b^{*}\right). \end{array}$$

In this study, a polynomial kernel of degree 2 was used for the SVM. As a global kernel, it allows data points that are far away from each other to also have an influence on the kernel values. The general polynomial kernel is given by

(9) $$\begin{array}{c} K\left(x,x_{i}\right)={\left(\left(x_{i}\cdot x\right)+1\right)}^{d}, \end{array}$$

where *d* is the order of the polynomial to be used. In this study, it was varied from 2 to 4. Higher orders were ignored because of a higher computational burden with no substantial gain in classification accuracy from our experience. 

### 2.4. Validation

**Figure 2:**
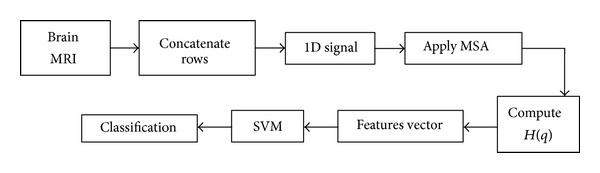
AD diagnosis system.

The design of the automated AD diagnosis system is shown in Figure 2. 

The validation experiments were conducted using the leave-one-out cross-validation method. Then, the average and standard deviation of the correct classification rate (CCR), sensitivity, and specificity were computed to evaluate the performance of the classifier. The three performance measures are defined as follows:

(10) $$\begin{array}{c} \text{CCR}=\frac{\text{Classified}\;\text{Samples}}{\text{Total}\;\text{Number}\;\text{of}\;\text{Samples}},\\ \text{Sensitivity}=\frac{\text{Correctly}\;\text{Classified}\;\text{Positive}\;\text{Samples}}{\text{True}\;\text{Positive}\;\text{Samples}},\\ \text{Specificity}=\frac{\text{Correctly}\;\text{Classified}\;\text{Negative}\;\text{Samples}}{\text{True}\;\text{Negative}\;\text{Samples}}, \end{array}$$

where positive samples and negative samples refer to AD and normal images, respectively. 

## 3. Results 

**Figure 3:**
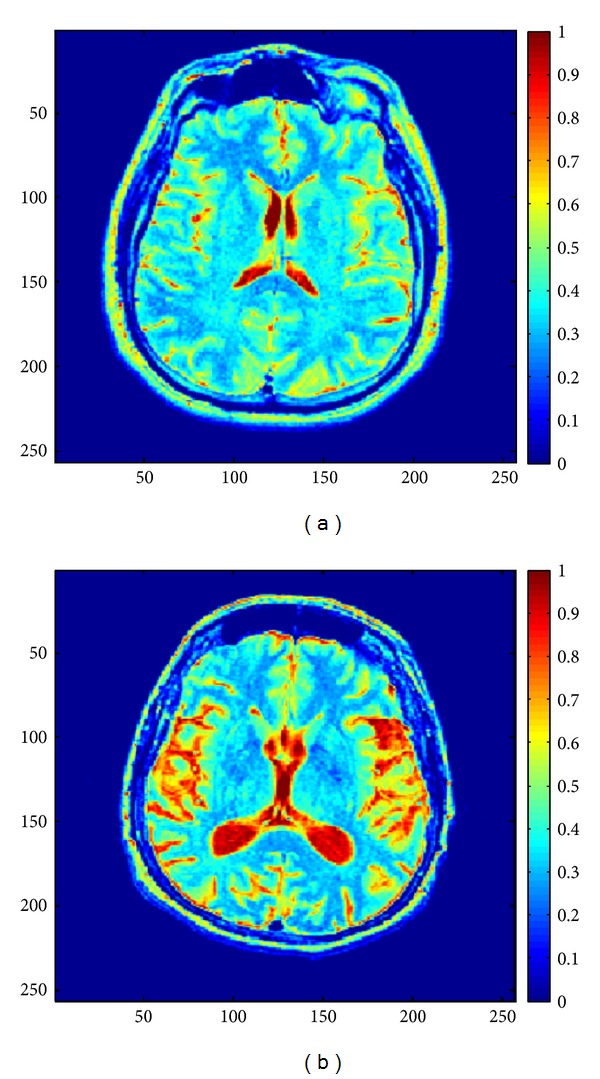
Healthy image (a) and AD image (b) in double color format.

**Figure 4:**
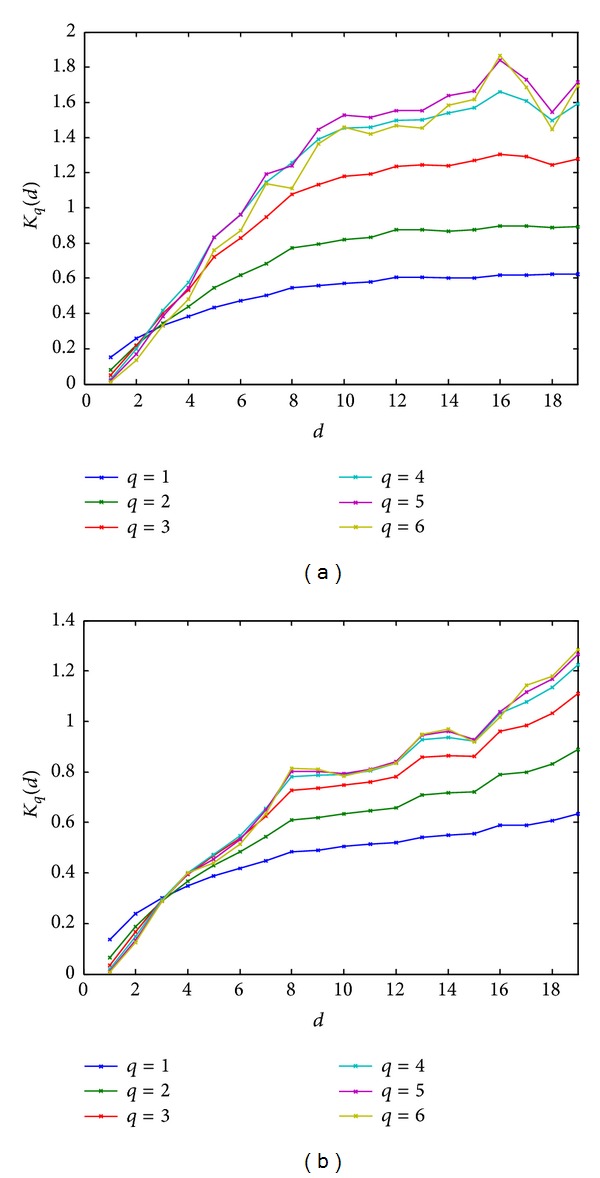
Multiscale analysis results of healthy image (a) and AD (b).

**Figure 5:**
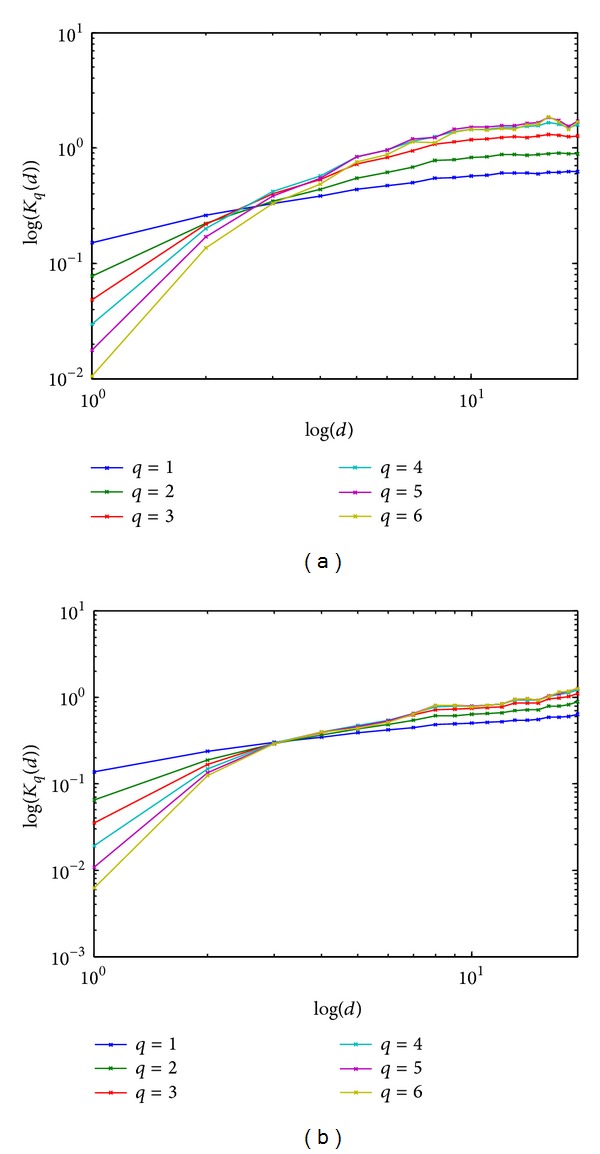
Multiscale analysis results on log-log scale of healthy image (a) and AD (b).

Before processing, the grayscale images as shown in Figure 1 are converted to double color format to perform MSA (see examples in Figure 3).  Figure 4 shows the obtained behavior of *K*_*q*_(*d*) as a function of *d* (see, (2)) for the images of healthy patients and those with AD and for *q* varying from 1 to 6, whereas *d* varies from 1 to 19.  Figure 5 provides the same information on a log-log scale.  Figure 4 reveals that, for the images of normal brains, *K*_*q*_(*d*) quickly reaches horizontal saturation for *q* = 1,2, 3; this is not so for the AD images where *K*_*q*_(*d*) increases monotonically for all *d*. In addition, the magnitude of *K*_*q*_(*d*) for a healthy image is in general larger than that of an AD image. In summary, Hurst's exponents appear to be different at each scale for the two types of brain MRIs. 

**Figure 6:**
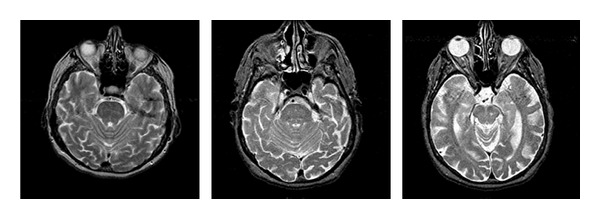
Examples of excluded AD images in the second experiment.

As mentioned previously, the order of the polynomial kernel used in the SVM was varied from 2 to 4. The best classification performance was obtained with a fourth-order kernel, for which the correct classification rate, sensitivity, and specificity were 99.18% ± 0.0083, 100%, and 98.20% ± 0.0182, respectively. A visual analysis of the AD images revealed that seventeen of them looked markedly different from the example in Figure 1 (Figure 6 shows three of them). Removing them from the validation database led to 100% classification accuracy by the SVM.

Finally, the MSA running time was 5.64 seconds, and the overall image processing time was about 8 seconds on a 3.30 GHz Core i5-2500 CPU using MATLAB 2012a codes, leading to an execution speed improvement of nearly two orders of magnitude in comparison to Hurst's exponent and DFA. 

## 4. Discussion and Conclusion 

The overall classification accuracy obtained in this work was 99.18% ± 0.01. However, excluding some atypical AD images led to 100% correct classification rate. This suggests that a multi-SVM approach, where one or more classifier handles the misclassified images by the first SVM, might lead to perfect classification accuracy in all situations. We intend to investigate this possibility. 

Our approach for AD detection outperformed most of the studies found in the literature, where the reported classification accuracy was between 86% and 96.9% [1–5, 7–13]. One exception is the work of [6] where perfect classification accuracy is reported. However, it is achieved with a database on only 45 MR images (25 healthy controls and 20 AD images), and the number of features used was higher than 1000 in comparison to the six used by our approach. We limited the number of features to six somewhat arbitrarily, and we have not investigated the effect of more or less features on the performance of the classifier. Future work should also investigate this. 

In summary, multiscale analysis-based Hurst's exponents were used for the classification of healthy brain images versus AD by a SVM with fourth-order kernel. The obtained results show the potential of using multiscale fractal analysis to differentiate healthy brain images from ones affected by Alzheimer's disease. The MSA algorithm took 5.64 seconds to analyze a brain MRI while the detrended fluctuation analysis (DFA) took 400 seconds in our previous work [12]. In addition, a larger database is used for validation. 

Finally, although we have obtained better result than the literature in general, it is difficult to draw definite conclusions since we used a different image database. In future work, we will explore a benchmark image depository such as the Alzheimer's Disease Neuroimaging Initiative (ADNI) database. Furthermore, we will investigate the effectiveness of MSA to classify AD images versus mild cognitive impairment (MCI). Indeed, the ability to correctly classify the AD and MCI images based on MSA Hurst's exponents might shed light on the ability to predict the conversion from MCI to AD, which is of clinical interest.
